# Prediction of catalytic residues using Support Vector Machine with selected protein sequence and structural properties

**DOI:** 10.1186/1471-2105-7-312

**Published:** 2006-06-21

**Authors:** Natalia V Petrova, Cathy H Wu

**Affiliations:** 1 Protein Information Resource, Department of Biochemistry and Molecular & Cellular Biology, Georgetown University Medical Center, Washington, DC 20007, USA

## Abstract

**Background:**

The number of protein sequences deriving from genome sequencing projects is outpacing our knowledge about the function of these proteins. With the gap between experimentally characterized and uncharacterized proteins continuing to widen, it is necessary to develop new computational methods and tools for functional prediction. Knowledge of catalytic sites provides a valuable insight into protein function. Although many computational methods have been developed to predict catalytic residues and active sites, their accuracy remains low, with a significant number of false positives. In this paper, we present a novel method for the prediction of catalytic sites, using a carefully selected, supervised machine learning algorithm coupled with an optimal discriminative set of protein sequence conservation and structural properties.

**Results:**

To determine the best machine learning algorithm, 26 classifiers in the WEKA software package were compared using a benchmarking dataset of 79 enzymes with 254 catalytic residues in a 10-fold cross-validation analysis. Each residue of the dataset was represented by a set of 24 residue properties previously shown to be of functional relevance, as well as a label {+1/-1} to indicate catalytic/non-catalytic residue. The best-performing algorithm was the Sequential Minimal Optimization (SMO) algorithm, which is a Support Vector Machine (SVM). The Wrapper Subset Selection algorithm further selected seven of the 24 attributes as an optimal subset of residue properties, with sequence conservation, catalytic propensities of amino acids, and relative position on protein surface being the most important features.

**Conclusion:**

The SMO algorithm with 7 selected attributes correctly predicted 228 of the 254 catalytic residues, with an overall predictive accuracy of more than 86%. Missing only 10.2% of the catalytic residues, the method captures the fundamental features of catalytic residues and can be used as a "catalytic residue filter" to facilitate experimental identification of catalytic residues for proteins with known structure but unknown function.

## Background

The high-throughput genome projects have resulted in a rapid accumulation of predicted protein sequences for a large number of organisms. Researchers have begun to systematically tackle protein functions and complex regulatory processes by studying organisms on a global scale, from genomes and proteomes to metabolomes and interactomes. Meanwhile, structural genomics projects have generated a growing number of protein structures of unknown function. To fully realize the value of these high-throughput data requires better understanding of protein function. With experimentally-verified information on protein function lagging behind, computational methods are needed for functional prediction of proteins. In particular, knowledge of the location of catalytic residues provides valuable insight into the mechanisms of enzyme-catalyzed reactions.

Many computational methods have been developed for predicting protein functions and functional residues involved in catalytic reactions, binding activities, and protein-protein interactions. Automated propagation of functional annotation from a protein with known function to homologous proteins is a well-established method for the assignment of protein function. However, reliable functional propagation generally requires a high degree of sequence similarity. For example, to transfer all four digits of an EC number at an error rate of below 10% needs at least 60% sequence identity [1], and only about 60% of the proteins can be annotated by a homology transfer of experimental functional information in 62 proteomes [2].

The evolutionary trace (ET) method is used for prediction of active sites and functional interfaces in proteins with known structure. Based on the observation that functional residues are more conserved than other residues, the method finds the most conserved residues at different sequence identity cutoffs and, as a final step, relies on human visual examination of the residues on protein structures [3]. While the ET method was shown successful in many case studies [4-6], the need for manual inspection in this original implementation is not suitable for automated large-scale analysis. Modified and automated versions of the ET method have been developed and tested on two protein datasets. In one study [7], the catalytic residues were predicted correctly for 62 (77.5%) out of 80 enzymes with the ACTSITE and SITE records from the PDB database [*1L*]; in another study [8], ~60% (79% by manual analysis) of catalytic residues were predicted correctly for 29 enzymes with experimentally characterized active sites.

Another group of methods, the *ab initio *methods [reviewed in [2,9]], do not use sequence conservation for functional site prediction. These methods exploit general protein properties, such as residue buffer capacity [10], the electrostatic energy of charged residues [11], protein subcellular localization [2], and conservation of local structural similarities [12,13]. These methods are potentially useful for the prediction of novel protein functions even if sequence conservation of the functional site in question is low.

The last group of methods combines sequence conservation with different aspects of protein structure [14-17]. Three-dimensional cluster analysis predicted functional residues by examination of spatially-adjacent conserved residues [14], and achieved a high recovery (83%) with low error rate (2%) for the prediction of catalytic residues in 15 enzymes. A similar method enriched with two additional structural parameters predicted ~47% of catalytic residues at the 5% false positive rate among 39 enzymes from the CDD database with manually curated catalytic sites [15]. A method for locating catalytic residues based on the sequence conservation, local special conservation, stability analysis, and geometrical location of the residue predicted 56% of catalytic residues in 49 enzymes [16]. The method considered only highly conserved D, E, K, R, H, S, T, N, Y, and C residues. A trained neural network (NN) with spatial clustering predicted over 69% of catalytic residues with a high false positive rate among 189 enzymes from the CATRES database [*2L*] containing manually curated catalytic residues [17]. The method used sequence conservation, residue type, and four structural parameters as inputs for the NN.

Direct comparison of methods is confounded by the use of different performance measures and different datasets of various size and quality. Nevertheless, the overall accuracy for the prediction of catalytic residues remains low (in the 70% range). This study aimed to develop an improved fully-automated method for the prediction of catalytic residues using a carefully selected, supervised machine learning algorithm coupled with an optimal discriminative set of protein sequence conservation and structural properties.

## Results and discussion

### Selection of the best machine learning algorithm using 24 residue properties

To determine the best machine learning algorithm for the predictive task, 26 classifiers currently available in the WEKA software package [[18], *3L*] were compared using their default parameters and a benchmarking dataset of 79 enzymes with 254 catalytic residues. The performance of the algorithms was measured by the Matthews correlation coefficients (MCC) in a 10-fold cross-validation analysis using three balanced datasets generated from the benchmarking data, each with an equal number of non-catalytic residues randomly chosen from all non-catalytic residues of the benchmarking dataset. Each residue was represented by a set of 24 sequence and structural attributes and a label of {+1/-1} to indicate whether the residue is catalytic (+1) or not (-1).

**Figure 1:**
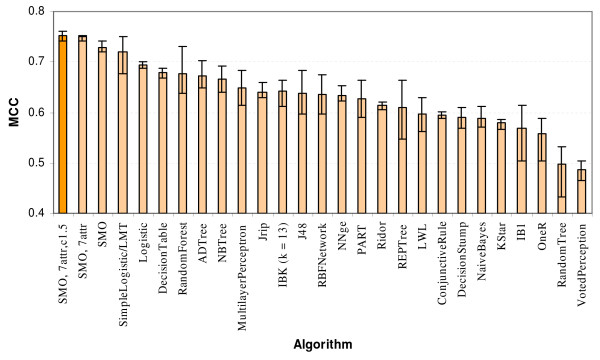
The performance of 26 machine learning algorithms for the prediction of catalytic residues as measured by the Matthews correlation coefficient (MCC) in 10-fold cross-validation analysis.

The best-performing algorithm was the *Sequential Minimal Optimization *(SMO) algorithm (Figure 1, *see "Methods" for detailed description*), which is a *Support Vector Machine *(SVM) [19]. The SVM is a learning machine for two-group classification problems that transforms the attribute space into multidimensional feature space using a kernel function to separate dataset instances by an optimal hyperplane [20]. The next three top algorithms are Simple Logistic/LMT, Logistic, and Decision Table, all containing automatic attribute selection for optimal performance.

### Selection of an optimal subset of residue properties for the SMO algorithm

**Figure 2:**
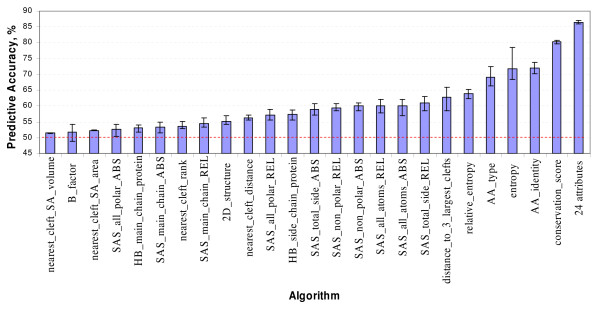
The predictive accuracy of the SMO algorithm based on individual residue properties in comparison with 24 combined attributes.

As SVM is sensitive to the presence of irrelevant attributes, proper attribute selection may further increase the accuracy of the SMO algorithm. Although relevant protein features for the prediction of catalytic residues are known, an optimal discriminative set of protein sequence conservation and structural properties has not been reported. To select an optimal subset of residue properties, we first analyzed how individual attributes from the initial set of 24 properties contributed to predictive accuracy. While the predictive accuracy with the combination of all 24 attributes reached 86%, the predictive potential of most individual attributes was significantly lower, many in the 50–60% ranges (Figure 2). The top five attributes all have to do with sequence conservation (*conservation_score, entropy, relative_entropy*) or amino acid identity (*AA_identity, AA_type*), with the *conservation_score *alone approaching 80% accuracy.

To determine the proper combination of attributes for the SMO classifier, we employed the Wrapper Subset Selection algorithm, which selects an optimal subset of attributes customized for a given classifier among all possible subsets of attributes [21]. Using a 10-fold cross-validation on three datasets, seven of the 24 attributes were selected as an optimal subset – namely, *conservation_score, AA_identity, HB_main_chain_protein, distance_to_3_largest_clefts, nearest_cleft_distance, nearest_cleft_rank*, and *nearest_cleft_SA_area *(Table 1). The four last features belong to one category of closely related attributes describing residue *relative position on protein surface; *whereas the first three belong to three independent attribute categories -*sequence conservation, residue identity*, and *hydrogen bonds *(*see "Methods"*). No further reduction of the set was possible, as the performance of SMO for all three datasets dropped if any of the seven attributes was eliminated. Consistent with the results in Figure 2, the removal of the *conservation_score *resulted in the most marked reduction (Table 1). Overall, the 7-attribute subset improved the SMO prediction using 24 attributes with a predictive accuracy from 86.38% to 87.42%, and MCC from 0.728 to 0.749.

Note that this is an optimal feature subset of the properties that provided best accuracy of the SMO algorithm in this study. This set does not necessarily represent the only suitable combination or all the relevant attributes. For example, *nearest_cleft_SA_area *can be substituted by the combination of *SAS_total_side_REL *and *nearest_cleft_SA_volume *attributes, resulting in another optimal subset of attributes [22].

### Analysis of the SMO prediction with the selected seven residue properties

**Figure 3:**
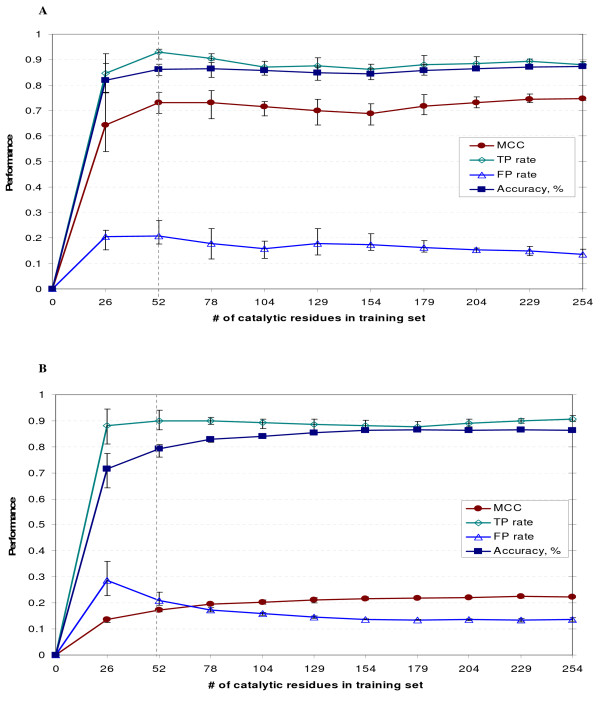
The learning curve of the SMO algorithm with the 7-attribute set in 10-fold cross-validation analysis using (**A**) a balanced dataset or (**B**) the whole benchmarking dataset as a test set.

With the seven selected attributes, the SMO algorithm correctly predicted 223 of the 254 catalytic residues (87.8% of true positives) with an overall predictive accuracy of more than 87% (Table 1). Since the benchmarking dataset had only 79 proteins, one may argue that the high performance of the SMO algorithm is a result of over-fitting the data, rather than a generalization of the classifier. To ensure that the accuracy is not attributable to the small size of the dataset, we further analyzed the learning curve of the algorithm using 10-fold cross-validation with four performance measures – MCC, % accuracy, true positive (TP) rate, and false positive (FP) rate. To measure the learning curve, we randomly split the data in each dataset into 10 parts and increased the size of the dataset by one part incrementally. The performance changed only slightly after 2/10 of the data (52 catalytic residues) were used (Figure 3).

As our benchmarking dataset consisted of structurally and functionally heterogeneous proteins *(see "Methods")*, this learning curve suggests that the enlargement of the dataset would not dramatically change the outcome of the prediction of the SMO algorithm, and that the algorithm and the selected features have captured the fundamental properties of catalytic residues (Figure 3A). A similar learning curve was obtained (for % accuracy, TP rate, and FP rate) using all 23,664 residues in the 79 proteins as a test set, except that the MCC curve was notably lower due to the large proportion of negative instances (Figure 3B and Table 2).

Since the selection of the optimal attribute subset was performed using balanced datasets, we compared the performance of the SMO algorithm on the entire benchmarking dataset. No significant changes in the performance of the SMO algorithm were detected after the reduction of the initial attribute set down to 7-attribute subset (Table 2). Therefore, the selected set of seven features is, in fact, optimal for the whole benchmarking dataset.

The evaluation on the whole benchmarking dataset mimics the performance of the SMO algorithm on the novel proteins, thus the SMO algorithm correctly predicted 228 of the 254 catalytic residues (89.8% of true positives) with an overall predictive accuracy of more than 86%.

Our result compared favorably with a feed-forward neural network (NN) trained using a scaled conjugate gradients algorithm (i.e., Multilayer Perceptron) to predict catalytic residues in 159 proteins from the CATRES database [17]. The comparison is limited to the performance measurements reported by authors: FP rate (Q_observed_), and MCC. The NN was trained on a dataset with 1:6 ratio and tested on a dataset with ~1:100 ratio between catalytic and non-catalytic residues, whereas our study was trained on a dataset of 1:1 ratio, and tested on datasets of 1:1 and 1:92 ratios (Table 2). The TP rate of our method is 0.90, whereas it is 0.56 before clustering (and 0.68 after clustering) for the NN. The MCC of our method is comparable with the MCC of the NN algorithm: SMO – 0.23, NN – 0.28 before clustering and 0.32 after clustering. The major differences between the two approaches are the selections of the attributes for residue representation and the machine learning algorithm. Note that the NN algorithm -'MultilayerPerceptron' was not among the top seven predictive algorithms in our initial study of best-performing machine learning methods (Figure 1). The parameters for the NN study were chosen based on the previous analysis of relevant features for the catalytic residues [23], such as conservation, diversity of position score, depth from surface, relative solvent accessibility, cleft colocalization, 2D structure, and amino acid identity, which collectively may not represent an optimal set.

## Conclusion

The analysis of the optimal subset selected from the initial 24 residue properties indicates that the SMO algorithm learns to distinguish catalytic from non-catalytic residues based on sequence conservation *(conservation_score)*, catalytic propensities of amino acids (*AA_identity*), relative position of the residue on protein surface (*distance_to_3_largest_clefts, nearest_cleft_distance, nearest_cleft_rank, nearest_cleft_SA_area*), and the number of hydrogen bonds between the residue main chain atoms and other atoms in the protein (*HB_main_chain_protein*). The SMO algorithm and the seven selected attributes seem to capture the fundamental features of catalytic residues, and can predict catalytic residues with accuracy > 86% for proteins with known structure.

This study shows that the choices of both machine learning algorithm and optimal attributes sets for the selected algorithm are critical for the prediction tasks. Conceivably, a similar approach can also be used for the prediction of binding site residues and residues involved in protein-protein interactions.

## Methods

### Overview

**Figure 4:**
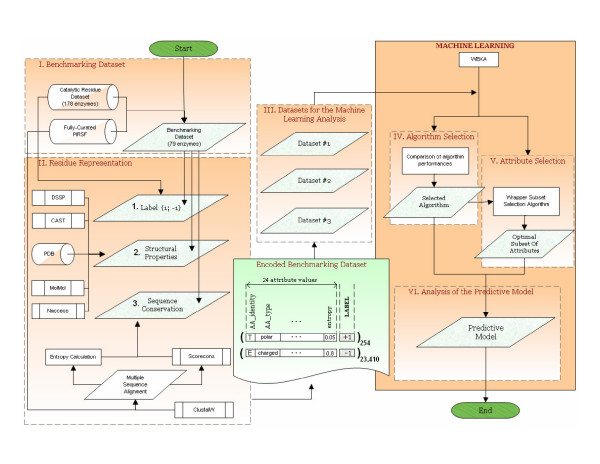
Method overview.

Figure 4 shows an overview of our method, which involves (i) compilation of benchmarking dataset, (ii) residue feature representation, (iii) creation of three datasets for machine learning analysis, (iv) selection of best-performing machine learning algorithm, (v) selection of an optimal subset of residue attributes, and (vi) analysis of the predictive model.

### Benchmarking dataset

The benchmarking dataset was compiled from the CATRES (Catalytic Residue Dataset) database [*2L*], which consisted of 615 manually-curated catalytic residues from 178 enzymes [23]. These catalytic sites were experimentally validated and manually collected from scientific literature based on a clear definition of catalytic residues. Catalytic residues in our study thus were defined the same as in CATRES. A subset of CATRES proteins in fully-curated PIRSF protein families [[24], *4L*] was used as the benchmarking data, which included 79 enzymes and 254 catalytic residues. Protein members in PIRSF families are homologous (sharing common ancestry) and homeomorphic (sharing full-length sequence similarity with common domain architecture).

The 79 enzymes in the benchmarking dataset are structurally and functionally heterogeneous based on SCOP fold classification [[25], *5L*], enzyme classification (EC number) [*6L*], and BLAST sequence similarity [26]. The fold classification indicates that 48.1% of these enzymes are in the α/β class, 30.4% belong to the α+β class, 10.1% each are assigned to mainly α and mainly β classes, and the remaining 1.3% belongs to the class of small proteins. According to the enzyme classification, the dataset has 79 (78 unique) EC numbers, including 20.5% oxidoreductases (EC 1.-.-.-), 25.6% transferases (EC 2), 28.2% hydrolases (EC 3), 18.0% lyases (EC 4), 2.6% isomerases (EC 5), and 5.1% ligases (EC 6). Note that two enzymes, 1e2a and 1gpr, belong to different structural classes (mainly α and mainly β classes, respectively), but have the same EC number (2.7.1.69) due to convergent evolution. Manual examination of the BLAST all-against-all search results and pairwise alignments of the 79 PDB-sequences of the enzymes revealed no sequence similarity among them.

The 79 proteins (identified by the PDB code) were: 1a26, 1a4i, 1a4s, 1ab8, 1ae7, 1afw, 1ah7, 1akm, 1aop, 1apx, 1apy, 1aq2, 1aw8, lb3r, lb57, lb93, 1bo1, 1brm, 1bs4, 1btl, 1bzy, 1cd5, 1chd, 1ctt, 1d4a, 1daa, 1dae, 1db3, 1dbt, 1dco, 1diz, 1dj0, 1dnk, 1dnp, 1dqs, 1dzr, 1e2a, 1ef8, 1eyi, 1fua, 1gim, 1gpm, 1gpr, 1grc, 1hxq, 1iph, 1jdw, 1kas, 1kra, 1lba, 11xa, 1mbb, 1mek, 1mla, 1moq, 1mpy, 1nba, 1nsp, 1pfk, 1pjb, 1pnl, 1pud, 1qfe, 1smn, 1uae, 1ula, 1uok, 1uox, 1wgi, 1xva, 2acy, 2alr, 2bbk, 2cpo, 2hgs, 2jcw, 2pfl, 2plc, 3eca.

### Feature representation of 24 residue properties

For the initial analysis, each residue of the benchmarking dataset was represented as a vector with 24 residue property values and a label {+1/-1} to indicate the catalytic (+1) and non-catalytic (-1) residue. The list of properties was chosen based mostly on the work of Bartlett et al. [23] and other authors who pointed out the possible relevance of particular residue properties [27,25]. This attribute set represents information about residue identity, sequence conservation, flexibility, solvent accessibility, relative position on protein surface, hydrogen bonds, and secondary structure (Table 3), as detailed below.

#### Residue identity

Amino acids have different propensities to be catalytic [23]. These propensities are captured by both the amino acid identity and amino acid types.

- *AA_identity: *A, C, D, E, F, G, H, I, K, L, M, N, P, Q, R, S, T, V, W, Y

- *AA_type: *The amino acids are grouped based on their chemico-physical properties into three types: charged (H, R, K, E, D), polar (Q, T, S, N, C, Y, W), and hydrophobic (G, F, L, M, A, I, P, V) [23].

#### Sequence conservation

A key property of catalytic residues is sequence conservation – they are generally more conserved than the rest of the protein. The residue conservation was calculated using the following three measures based on multiple sequence alignments of the respective PIRSF protein family generated by ClustalW [29].

- *entropy: *The Shannon entropy represents conservation in a range from 0 to 1, where 0 means strict conservation. At each position in the sequence alignment, entropy was estimated using the 9-Component Dirichlet Mixture algorithm [30]. This algorithm takes into account not only actual occurrences of amino acids in the position, but also the amino acid context, thus increasing chances for amino acids with similar biochemical properties to be observed in the same position [31]. The gap probability is assigned to l/(number of sequences in the multiple sequence alignment).

- *relative_entropy: *This was calculated as a proportion to the highest entropy of the multiple sequence alignment for each protein family. Note that the highest position entropy was chosen among all positions in which the entropy value was not an outlier.

- *conservation_score*: The Scorecons server [32] was used to calculate the conservation score with the default scoring method and parameters. The method assigns a score for each position in the sequence alignment using a modified PET91 matrix and sequence weighting that normalizes the alignment against sequence redundancy. The conservation score varies between 0 and 1, with 1 being the most conserved.

#### Flexibility

Several studies revealed the importance of local or even global flexibility of the protein structure for proper functioning. A flexible structure may allow a protein to bind to many partners or to achieve low affinity with high specificity by structural rearrangement upon binding [27,33].

- *B-factor *was calculated as a sum of all atomic B-factors of the residue from PDB.

#### Solvent accessibility

The surface area is important because interaction with other molecules happens on the surface. 89% of catalytic residues have solvent accessibility less than 30%, but it is increased upon binding of the enzyme with its ligand [23]. Different aspects of the solvent accessible surface (SAS) of biologically active chains from PDB were calculated using the Naccess program [34] with the default setting. Naccess uses Lee and Richards's method [35] to calculate the solvent accessible area of a group of atoms or of a whole residue for a protein. The default radius of a rolling probe is 1.4 Å, which imitates the size of a water molecule. A residue solvent accessible area is calculated as (i) a sum of solvent accessible areas for each defined group of atoms, labeled as ***ABS***, and (ii) as a % of accessibility compared to the accessibility of that residue type in an extended ALA-x-ALA tripeptide, labeled as ***REL***. The solvent accessibility is represented by the following ten attributes:

- *SAS_all_atoms_ABS/REL: *SAS was calculated for all residue atoms.

- *SAS_total_side_ABS/REL: *SAS was calculated for the side-chain atoms of the residue, including C_α_, so that glycine would have a side-chain accessibility.

- *SAS_main_chain_ABS/REL*: SAS for the main chain atoms of the residue, excluding C_α_.

- *SAS_non_polar_ABS/REL*: SAS for non-polar side chain atoms was calculated for all non-oxygen and non-nitrogen atoms in the side-chain of the residue.

- *SAS_all_polar_ABS/REL*: SAS for all oxygen and nitrogen atoms in the side-chain of the residue.

#### Relative position on protein surface

Enzyme active sites are usually located in large and deep protein clefts [28]. It was observed that at least one catalytic residue is located in a cleft for 93% of proteins, and that 85% of catalytic residues are located in the three largest clefts on the protein surface [23]. Several attributes were used to represent the relative position on protein surface based on the output of the CASTp server [[36], *8L*] for biologically active chain from PDB. Since atoms of the same residue can be in different clefts, the cleft number is the largest cleft for a given residue. CASTp numeration of the clefts starts with the smallest one first, so we reversed the numbering so that the largest cleft of the protein would be the first cleft of the protein. If a residue was not part of any cleft, the cleft number was assigned zero. The attributes include:

- *nearest_cleft_rank, nearest_cleft_SA_volume, nearest_cleft_SA_area: *the three attributes for rank, solvent accessible volume and solvent accessible area are obtained directly from the output of the CASTp server.

- *nearest_cleft_distance: *The distance to the nearest cleft was calculated as a minimal distance between any atom (except hydrogen) of the residue and any atom (except hydrogen) of the residues of the closest cleft. If a residue was in a cleft, the distance was assigned zero.

- *distance_to_3_largest_clefts *was calculated as a minimal distance between any atom (except hydrogens) of the residue and any atom (except hydrogens) of the residues of the 3 largest clefts. If a residue was a part of the 3 largest clefts, the distance was assigned zero.

#### Hydrogen bonds

The majority of catalytic residues participate in hydrogen bonding through their main chain [23]. Two attributes relating to hydrogen bonding were calculated using the MolMol program [37], a molecular graphics program for display, analysis, and manipulation of three-dimensional structures of biological macromolecules. The attributes are:

- *HB_main_chain_protein, HB_side_chain_protein: *number of hydrogen bonds of the residue atoms from the main chain or side chain, respectively, with any other atom in the protein.

#### Secondary structure

It was observed that about half of catalytic residues are localized in the coiled regions of the protein [23]. The attribute is:

- *2D_structure*: the 2D structure of individual residues was based on the DSSP program, which assigns a single letter code (H, E, S, T, C, G, B, I, -) to represent different 2D structure types [38].

### Feature encoding

Each residue was represented as a vector with attribute values and a label indicating the catalytic (+1) and non-catalytic (-1) residue. Every attribute was represented by one unit: a character (AA_identity, 2D_structure), string (AA_type), or a real number (the rest of the attributes).

### Datasets for machine learning analysis

The selection, training, and evaluation of the machine learning algorithms were performed using three datasets derived from the benchmarking dataset after feature encoding. A residue was excluded in the datasets for machine learning analysis if it was a non-trivial amino acids (e.g., B, X, Z) or it was deemed an outlier based on the interquartile range [39] of the entropy values for the given protein. The outliers were usually present in regions of the multiple sequence alignment with large numbers of gaps.

The processing resulted in a total of 23,664 residues from the benchmarking dataset of 79 enzymes, including 254 catalytic and 23,410 non-catalytic residues (1:92). Since the fraction of catalytic residues in the dataset was small, we created three balanced datasets (1:1), each containing an equal number of negatively labeled instances (non-catalytic residues) and positively labeled ones (catalytic residues). Thus, each dataset has all 254 catalytic residues and the equivalent number of non-catalytic ones, randomly chosen from the 23,410 non-catalytic residues.

### Machine learning

The selections of the best-performing algorithm and an optimal set of properties for the selected algorithm were performed using WEKA (Waikato Environment for Knowledge Analysis). WEKA is a JAVA software package from the University of Waikato, New Zealand [[18], *3L*] with an open source issued under the GNU General Public License. The package provides a collection of machine learning algorithms for data mining tasks. The algorithms can either be applied directly to a dataset or called from the user's own Java code. WEKA contains tools for data pre-processing, classification, regression, clustering, association rules and visualization, and is well suited for developing new machine learning schemes. In this study, all algorithms were trained using WEKA's default settings, except in the IBK algorithm where the parameter K was chosen to be 13 to maximize the algorithm's performance.

#### Support Vector Machine classifier – Sequential Minimal Optimization (SMO)

The WEKA's implementation of SMO converts all nominal attributes into binary ones and normalizes all attributes by default. We used the default polynomial kernel function for the analysis with default parameters, such as the complexity parameter C = 1.0, exponent = 1.0.

### Performance measure

The performance of each algorithm was measured as an average value in a 10-fold cross-validation analysis, where each dataset was divided into 10 parts – 9 parts for model learning (training) and the remaining part for validation (testing). Four performance measures were used: Matthews Correlation Coefficient (MCC) [40], true positive (TP) rate (for sensitivity), false positive (FP) rate (for selectivity), and predictive accuracy, as defined below.

MCC=(TpTn−FpFn)(Tp+Fp)(Tp+Fn)(Tn+Fn)(Tn+Fp)=(TpTn−FpFn)P^ P×N^ N={0,guessin⁡g1,all correct
 MathType@MTEF@5@5@+=feaafiart1ev1aaatCvAUfKttLearuWrP9MDH5MBPbIqV92AaeXatLxBI9gBaebbnrfifHhDYfgasaacH8akY=wiFfYdH8Gipec8Eeeu0xXdbba9frFj0=OqFfea0dXdd9vqai=hGuQ8kuc9pgc9s8qqaq=dirpe0xb9q8qiLsFr0=vr0=vr0dc8meaabaqaciaacaGaaeqabaqabeGadaaakeaacqWGnbqtcqWGdbWqcqWGdbWqcqGH9aqpdaWcaaqaaiabcIcaOiabdsfaujabdchaWjabdsfaujabd6gaUjabgkHiTiabdAeagjabdchaWjabdAeagjabd6gaUjabcMcaPaqaamaakaaabaGaeiikaGIaemivaqLaemiCaaNaey4kaSIaemOrayKaemiCaaNaeiykaKIaeiikaGIaemivaqLaemiCaaNaey4kaSIaemOrayKaemOBa4MaeiykaKIaeiikaGIaemivaqLaemOBa4Maey4kaSIaemOrayKaemOBa4MaeiykaKIaeiikaGIaemivaqLaemOBa4Maey4kaSIaemOrayKaemiCaaNaeiykaKcaleqaaaaakiabg2da9maalaaabaGaeiikaGIaemivaqLaemiCaaNaemivaqLaemOBa4MaeyOeI0IaemOrayKaemiCaaNaemOrayKaemOBa4MaeiykaKcabaWaaOaaaeaacuWGqbaugaqcaiabbccaGiabdcfaqjabgEna0kqbd6eaozaajaGaeeiiaaIaemOta4ealeqaaaaakiabg2da9maaceaabaqbaeqabiGaaaqaaiabicdaWiabcYcaSaqaaiabdEgaNjabdwha1jabdwgaLjabdohaZjGbcohaZjabcMgaPjabc6gaUjabdEgaNbqaaiabigdaXiabcYcaSaqaaiabdggaHjabdYgaSjabdYgaSjabbccaGiabdogaJjabd+gaVjabdkhaYjabdkhaYjabdwgaLjabdogaJjabdsha0baaaiaawUhaaaaa@9248@

FP rate=Fp(Tn+Fp)=FpN=(1−specificity)=>probability of incorrectly predicting negatives
 MathType@MTEF@5@5@+=feaafiart1ev1aaatCvAUfKttLearuWrP9MDH5MBPbIqV92AaeXatLxBI9gBaebbnrfifHhDYfgasaacH8akY=wiFfYdH8Gipec8Eeeu0xXdbba9frFj0=OqFfea0dXdd9vqai=hGuQ8kuc9pgc9s8qqaq=dirpe0xb9q8qiLsFr0=vr0=vr0dc8meaabaqaciaacaGaaeqabaqabeGadaaakeaacqWGgbGrieGacqWFqbaucqqGGaaicqWFYbGCcqWGHbqycqWG0baDcqWGLbqzcqGH9aqpdaWcaaqaaiabdAeagjabdchaWbqaaiabcIcaOiabdsfaujabd6gaUjabgUcaRiabdAeagjabdchaWjabcMcaPaaacqGH9aqpdaWcaaqaaiabdAeagjabdchaWbqaaiabd6eaobaacqGH9aqpcqGGOaakcqaIXaqmcqGHsislcqqGZbWCcqqGWbaCcqqGLbqzcqqGJbWycqqGPbqAcqqGMbGzcqqGPbqAcqqGJbWycqqGPbqAcqqG0baDcqqG5bqEcqqGPaqkcqGH9aqpcqGH+aGpcqqGWbaCcqqGYbGCcqqGVbWBcqqGIbGycqqGHbqycqqGIbGycqqGPbqAcqqGSbaBcqqGPbqAcqqG0baDcqqG5bqEcqqGGaaicqqGVbWBcqqGMbGzcqqGGaaicqqGPbqAcqqGUbGBcqqGJbWycqqGVbWBcqqGYbGCcqqGYbGCcqqGLbqzcqqGJbWycqqG0baDcqqGSbaBcqqG5bqEcqqGGaaicqqGWbaCcqqGYbGCcqqGLbqzcqqGKbazcqqGPbqAcqqGJbWycqqG0baDcqqGPbqAcqqGUbGBcqqGNbWzcqqGGaaicqqGUbGBcqqGLbqzcqqGNbWzcqqGHbqycqqG0baDcqqGPbqAcqqG2bGDcqqGLbqzcqqGZbWCaaa@98BB@

TP rate=Tp(Tp+Fn)=TpP=sensitivity=>probability of correctly predicting positives
 MathType@MTEF@5@5@+=feaafiart1ev1aaatCvAUfKttLearuWrP9MDH5MBPbIqV92AaeXatLxBI9gBaebbnrfifHhDYfgasaacH8akY=wiFfYdH8Gipec8Eeeu0xXdbba9frFj0=OqFfea0dXdd9vqai=hGuQ8kuc9pgc9s8qqaq=dirpe0xb9q8qiLsFr0=vr0=vr0dc8meaabaqaciaacaGaaeqabaqabeGadaaakeaacqWGubavieGacqWFqbaucqqGGaaicqWFYbGCcqWGHbqycqWG0baDcqWGLbqzcqGH9aqpdaWcaaqaaiabdsfaujabdchaWbqaaiabcIcaOiabdsfaujabdchaWjabgUcaRiabdAeagjabd6gaUjabcMcaPaaacqGH9aqpdaWcaaqaaiabdsfaujabdchaWbqaaiabdcfaqbaacqGH9aqpcqqGZbWCcqqGLbqzcqqGUbGBcqqGZbWCcqqGPbqAcqqG0baDcqqGPbqAcqqG2bGDcqqGPbqAcqqG0baDcqqG5bqEcqGH9aqpcqGH+aGpcqqGWbaCcqqGYbGCcqqGVbWBcqqGIbGycqqGHbqycqqGIbGycqqGPbqAcqqGSbaBcqqGPbqAcqqG0baDcqqG5bqEcqqGGaaicqqGVbWBcqqGMbGzcqqGGaaicqqGJbWycqqGVbWBcqqGYbGCcqqGYbGCcqqGLbqzcqqGJbWycqqG0baDcqqGSbaBcqqG5bqEcqqGGaaicqqGWbaCcqqGYbGCcqqGLbqzcqqGKbazcqqGPbqAcqqGJbWycqqG0baDcqqGPbqAcqqGUbGBcqqGNbWzcqqGGaaicqqGWbaCcqqGVbWBcqqGZbWCcqqGPbqAcqqG0baDcqqGPbqAcqqG2bGDcqqGLbqzcqqGZbWCaaa@9367@

Accuracy=Tp+Tn(Tp+Fn+Tn+Fp)×100%=Tp+TnP+N×100%={X%,guessin⁡g100%,all correctwhereX=majority class procent
 MathType@MTEF@5@5@+=feaafiart1ev1aaatCvAUfKttLearuWrP9MDH5MBPbIqV92AaeXatLxBI9gBaebbnrfifHhDYfgasaacH8akY=wiFfYdH8Gipec8Eeeu0xXdbba9frFj0=OqFfea0dXdd9vqai=hGuQ8kuc9pgc9s8qqaq=dirpe0xb9q8qiLsFr0=vr0=vr0dc8meaabaqaciaacaGaaeqabaqabeGadaaakeaacqWGbbqqcqWGJbWycqWGJbWycqWG1bqDcqWGYbGCcqWGHbqycqWGJbWycqWG5bqEcqGH9aqpdaWcaaqaaiabdsfaujabdchaWjabgUcaRiabdsfaujabd6gaUbqaaiabcIcaOiabdsfaujabdchaWjabgUcaRiabdAeagjabd6gaUjabgUcaRiabdsfaujabd6gaUjabgUcaRiabdAeagjabdchaWjabcMcaPaaacqGHxdaTcqaIXaqmcqaIWaamcqaIWaamcqGGLaqjcqGH9aqpdaWcaaqaaiabdsfaujabdchaWjabgUcaRiabdsfaujabd6gaUbqaaiabdcfaqjabgUcaRiabd6eaobaacqGHxdaTcqaIXaqmcqaIWaamcqaIWaamcqGGLaqjcqGH9aqpdaGabaqaauaabaqadiaaaeaacqWGybawcqGGLaqjcqGGSaalaeaacqWGNbWzcqWG1bqDcqWGLbqzcqWGZbWCcyGGZbWCcqGGPbqAcqGGUbGBcqWGNbWzaeaacqaIXaqmcqaIWaamcqaIWaamcqGGLaqjcqGGSaalaeaacqWGHbqycqWGSbaBcqWGSbaBcqqGGaaicqWGJbWycqWGVbWBcqWGYbGCcqWGYbGCcqWGLbqzcqWGJbWycqWG0baDaeaacqWG3bWDcqWGObaAcqWGLbqzcqWGYbGCcqWGLbqzaeaacqWGybawcqGH9aqpcqWGTbqBcqWGHbqycqWGQbGAcqWGVbWBcqWGYbGCcqWGPbqAcqWG0baDcqWG5bqEcqqGGaaicqWGJbWycqWGSbaBcqWGHbqycqWGZbWCcqWGZbWCcqqGGaaicqWGWbaCcqWGYbGCcqWGVbWBcqWGJbWycqWGLbqzcqWGUbGBcqWG0baDaaaacaGL7baaaaa@AC3B@

Where Tp, Fp, Tn, Fn, P, N, P^
 MathType@MTEF@5@5@+=feaafiart1ev1aaatCvAUfKttLearuWrP9MDH5MBPbIqV92AaeXatLxBI9gBaebbnrfifHhDYfgasaacH8akY=wiFfYdH8Gipec8Eeeu0xXdbba9frFj0=OqFfea0dXdd9vqai=hGuQ8kuc9pgc9s8qqaq=dirpe0xb9q8qiLsFr0=vr0=vr0dc8meaabaqaciaacaGaaeqabaqabeGadaaakeaacuWGqbaugaqcaaaa@2DE5@, and N^
 MathType@MTEF@5@5@+=feaafiart1ev1aaatCvAUfKttLearuWrP9MDH5MBPbIqV92AaeXatLxBI9gBaebbnrfifHhDYfgasaacH8akY=wiFfYdH8Gipec8Eeeu0xXdbba9frFj0=OqFfea0dXdd9vqai=hGuQ8kuc9pgc9s8qqaq=dirpe0xb9q8qiLsFr0=vr0=vr0dc8meaabaqaciaacaGaaeqabaqabeGadaaakeaacuWGobGtgaqcaaaa@2DE1@ represent the number of residues that are true positives, false positives, true negatives, false negatives, labeled as positives/negatives in a dataset, and predicted as positives/negatives by classifier, respectively.

The FP rate and TP rate can be used for comparison of the results with different positive-to-negative ratios, whereas accuracy and MCC are sensitive to dataset imbalance.

## Abbreviations

2D, secondary; Å, angstrom; AA, amino acid; ABS, ABSolute; ADTree, Alternating Decision Tree; CASTp, Computed Atlas of Surface Topography of proteins; CDD, Conserved Domain Database; DSSP, Database of Secondary Structure in Proteins; EC, Enzyme Commission; ET, Evolutionary Trace; Fn (FN), False negatives; Fp (FP), False positives; HB, Hydrogen Bond; IB1, nearest-neighbor classifier; IBK, K-nearest neighbors classifier; J48, a pruned C4.5 decision tree; Jrip, a propositional rule learner, Repeated Incremental Pruning to Produce Error Reduction (RIPPER); LMT, Logistic Model Trees; LWL, Locally Weighted Learning algorithm; MCC, Matthews Correlation Coefficient; MolMol, MOLecule analysis and MOLecule display; NBTree, decision Tree with Naive Bayes classifiers at the leaves; NN, neural network; NNge, Nearest-Neighbor-like algorithm using non-nested generalized exemplars; OneR, One-Rule classifier; PART, a partial C4.5 decision tree algorithm; PDB, Protein Data Bank; PIR, Protein Information Resources Database; RBFNetwork, a normalized Gaussian radial basis function network; REL, relevant; REPTree, a decision tree learner; Ridor, RIpple-DOwn Rule learner; SA, solvent accessible; SAS, Solvent Accessible Surface; Scorecons, Score Conservation; SMO, Sequential Minimal Optimization; SVM, Support Vector Machine; Tn (TN), True negative; Tp (TP), True positive; WEKA, Waikato Environment for Knowledge Analysis.

## Authors' contributions

NP designed the analysis, developed the source code, conducted the study, and wrote the manuscript. CW coordinated the study, helped drafting the manuscript, and critically revised its content. All authors read and approve of the final manuscript.

## Links

1L. **The Protein Data Bank (PDB)**



2L. **Catalytic Residue Dataset database (CATRES)**



3L. **Waikato Environment for Knowledge Analysis (WEKA)**



4L. **PIRSF protein family database**



5L. **Structural Classification of Proteins (SCOP)**



6L. **Enzyme Nomenclature **(EC)



7L. **The Scorecons server**



8L. **The CASTp server, Computed Atlas of Surface Topography of proteins**



## Figures and Tables

**Table 1:** Performance of the SMO classifier in the absence of individual residue property in the optimal 7-attribute set in 10-fold cross-validation analysis

**Attribute**	**MCC in the absence of the attribute**				**Predictive accuracy in the absence of the attribute, %**			
	
	**DATASET**			**Average**	**DATASET**			**Average**
	***1* ^*st*^**	***2* ^*nd*^**	***3* ^*rd*^**		***1* ^*st*^**	***2* ^*nd*^**	***3* ^*rd*^**	

1. conservation_score	0.526	0.536	0.483	0.515	76.17	76.52	73.97	75.55
2. AA_identity	0.668	0.679	0.660	0.669	83.40	83.95	82.97	83.44
3. nearest_cleft_distance	0.708	0.746	0.707	0.720	85.35	87.28	85.32	85.98
4. distance_to_3_largest_clefts	0.724	0.757	0.726	0.736	86.13	87.87	86.30	86.77
5. HB_main_chain_protein	0.725	0.746	0.738	0.736	86.13	87.28	86.89	86.77
6. nearest_cleft_rank	0.740	0.746	0.730	0.739	86.91	87.28	86.50	86.90
7. nearest_cleft_SA_area	0.736	0.746	0.738	0.740	86.72	87.28	86.89	86.96

all attributes (24)	**0.720**	**0.722**	**0.742**	**0.728**	**85.94**	**86.11**	**87.08**	**86.38**
selected attributes (7)	**0.752**	**0.753**	**0.742**	**0.749**	**87.50**	**87.67**	**87.08**	**87.42**

**Table 2:** The properties and performance of two test datasets: a balanced dataset and whole benchmarking dataset

	**TEST SET**	**BALANCED**		**BENCHMARKING**	
**TRAINING SET PARAMETERS**	**Number of catalytic residues**	254×910* MathType@MTEF@5@5@+=feaafiart1ev1aaatCvAUfKttLearuWrP9MDH5MBPbIqV92AaeXatLxBI9gBaebbnrfifHhDYfgasaacH8akY=wiFfYdH8Gipec8Eeeu0xXdbba9frFj0=OqFfea0dXdd9vqai=hGuQ8kuc9pgc9s8qqaq=dirpe0xb9q8qiLsFr0=vr0=vr0dc8meaabaqaciaacaGaaeqabaqabeGadaaakeaacqaIYaGmcqaI1aqncqaI0aancqGHxdaTdaWcaaqaaiabiMda5aqaaiabigdaXiabicdaWaaacqGGQaGkaaa@356D@	254×910* MathType@MTEF@5@5@+=feaafiart1ev1aaatCvAUfKttLearuWrP9MDH5MBPbIqV92AaeXatLxBI9gBaebbnrfifHhDYfgasaacH8akY=wiFfYdH8Gipec8Eeeu0xXdbba9frFj0=OqFfea0dXdd9vqai=hGuQ8kuc9pgc9s8qqaq=dirpe0xb9q8qiLsFr0=vr0=vr0dc8meaabaqaciaacaGaaeqabaqabeGadaaakeaacqaIYaGmcqaI1aqncqaI0aancqGHxdaTdaWcaaqaaiabiMda5aqaaiabigdaXiabicdaWaaacqGGQaGkaaa@356D@	254	254
	**Catalytic vs. Non-catalytic Ratio**	1:1	1:1	1:1	1:1
	**Number of attributes used**	**24**	**7**	**24**	**7**

**TEST SET PARAMETERS**	**Number of catalytic residues**	254×110* MathType@MTEF@5@5@+=feaafiart1ev1aaatCvAUfKttLearuWrP9MDH5MBPbIqV92AaeXatLxBI9gBaebbnrfifHhDYfgasaacH8akY=wiFfYdH8Gipec8Eeeu0xXdbba9frFj0=OqFfea0dXdd9vqai=hGuQ8kuc9pgc9s8qqaq=dirpe0xb9q8qiLsFr0=vr0=vr0dc8meaabaqaciaacaGaaeqabaqabeGadaaakeaacqaIYaGmcqaI1aqncqaI0aancqGHxdaTdaWcaaqaaiabigdaXaqaaiabigdaXiabicdaWaaacqGGQaGkaaa@355D@	254×110* MathType@MTEF@5@5@+=feaafiart1ev1aaatCvAUfKttLearuWrP9MDH5MBPbIqV92AaeXatLxBI9gBaebbnrfifHhDYfgasaacH8akY=wiFfYdH8Gipec8Eeeu0xXdbba9frFj0=OqFfea0dXdd9vqai=hGuQ8kuc9pgc9s8qqaq=dirpe0xb9q8qiLsFr0=vr0=vr0dc8meaabaqaciaacaGaaeqabaqabeGadaaakeaacqaIYaGmcqaI1aqncqaI0aancqGHxdaTdaWcaaqaaiabigdaXaqaaiabigdaXiabicdaWaaacqGGQaGkaaa@355D@	254	254
	**Catalytic vs. Non-catalytic Ratio**	1:1	1:1	1:92	1:92
	**Number of attributes used**	**24**	**7**	**24**	**7**

**PERFORMANCE**	***Accuracy, %***	86.38	87.42	86.68	86.96
	***TP rate***	0.88	0.89	0.90	0.90
	***FP rate***	0.15	0.14	0.13	0.13
	***MCC***	0.73	0.75	0.23	0.23

*- *number of catalytic residues in each fold in 10-fold cross- validation analysis*

**Table 3:** The initial set of 24 residue properties

**#**	**ATTRIBUTE**	**PROGRAM/DATABASE USED**
**Residue Identity**		

1.	AA_identity	PDB database [41, *1L*]
2.	AA_type	[23]

**Sequence Conservation**		

3.	entropy	9-Component Dirichlet
4.	relative_entropy	Mixture algorithm [30]
5.	conservation_score	Scorecons server [32, *7L*]

**Flexibility**		

6.	B_factor	PDB database [41, *1L*]

**Solvent Accessibility**		

7.	SAS_all_atoms_ABS	Naccess program [34]
8.	SAS_all_atoms_REL	
9.	SAS_total_side_ABS	
10.	SAS_total_side_REL	
11.	SAS_main_chain_ABS	
12.	SAS_main_chain_REL	
13.	SAS_non_polar_ABS	
14.	SAS_non_polar_REL	
15.	SAS_all_polar_ABS	
16.	SAS_all_polar_REL	

	**Relative Position on protein Surface**	

17.	nearest_cleft_rank	CASTp server [36, *8L*],
18.	nearest_cleft_SA_volume	PDB database [41, *1L*]
19.	nearest_cleft_SA_area	
20.	nearest_cleft_distance	
21.	distance_to_3_largest_clefts	

	**Hydrogen Bonds**	

22.	HB_main_chain_protein	MolMol Program [37]
23.	HB_side_chain_protein	

	**Secondary Structure**	

24.	2D_structure	DSSP program [38]
